# Fluconazolium picrate

**DOI:** 10.1107/S1600536810036329

**Published:** 2010-09-15

**Authors:** Grzegorz Dutkiewicz, C. S. Chidan Kumar, H. S. Yathirajan, B. Narayana, Maciej Kubicki

**Affiliations:** a Department of Chemistry, Adam Mickiewicz University, Grunwaldzka 6, 60-780 Poznań, Poland; b Department of Studies in Chemistry, University of Mysore, Manasagangotri, Mysore 570 006, India; c Department of Studies in Chemistry, Mangalore University, Mangalagangotri 574 199, India

## Abstract

The title compound, C_13_H_13_F_2_N_6_O^+^·C_6_H_2_N_3_O_7_^−^, is the first structurally characterized salt of the cation of fluconazole [systematic name 2-(2,4-difluorophenyl)-1,3-bis(1*H*-1,2,4-triazol-1-yl)propan-2-ol], a synthetic anti­fungal agent. In the crystal, the components are linked by O—H⋯O hydrogen bonding between the hy­droxy group of the fluconazolium cation and the C=O(−) group of the picrate anion. This complex is additionally stabilized by secondary, but relatively short, C—H⋯O inter­actions. The dimers thus formed are connected by N—H⋯N cation–cation hydrogen bonds into helices running along [010]. Neighboring helices of opposite handedness are joined by weak anion–anion C—H⋯O(nitro) inter­actions. In the cation, the mean planes of the three rings are approximately, within *ca* 25°, parallel to the central C—O bond. In the picrate anion two nitro groups, in turn, are almost coplanar with the ring plane [forming dihedral angles of 6.5 (2) and 3.8 (2)°] while the third nitro group is significantly twisted [by 46.79 (13)°].

## Related literature

For the anti­fungal properties of fluconazole, see: Brammer *et al.* (1990 ▶); Caira *et al.* (2003 ▶). For a description of the Cambridge Structural Database, see: Allen (2002 ▶).
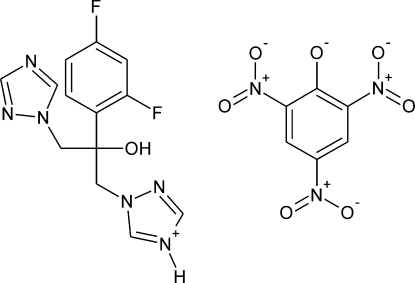


## Experimental

### 

#### Crystal data

C_13_H_13_F_2_N_6_O^+^·C_6_H_2_N_3_O_7_^−^
*M* *_r_* = 535.40
Monoclinic, 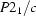

*a* = 5.647 (1) Å
*b* = 17.347 (2) Å
*c* = 22.571 (2) Å
β = 94.27 (1)°
*V* = 2204.9 (5) Å^3^
*Z* = 4
Mo *K*α radiation
μ = 0.14 mm^−1^
*T* = 100 K
0.2 × 0.2 × 0.15 mm

#### Data collection

Kuma KM-4-CCD four-circle diffractometer
18260 measured reflections
5165 independent reflections
3576 reflections with *I* > 2σ(*I*)
*R*_int_ = 0.023

#### Refinement

*R*[*F* ^2^ > 2σ(*F* ^2^)] = 0.035
*wR*(*F* ^2^) = 0.082
*S* = 0.99
5165 reflections
403 parameters
All H-atom parameters refined
Δρ_max_ = 0.25 e Å^−3^
Δρ_min_ = −0.24 e Å^−3^

### 

Data collection: *CrysAlis PRO* (Oxford Diffraction, 2009 ▶); cell refinement: *CrysAlis PRO*; data reduction: *CrysAlis PRO*; program(s) used to solve structure: *SIR92* (Altomare *et al.*, 1993 ▶); program(s) used to refine structure: *SHELXL97* (Sheldrick, 2008 ▶); molecular graphics: *Stereochemical Workstation Operation Manual* (Siemens, 1989 ▶); software used to prepare material for publication: *SHELXL97*.

## Supplementary Material

Crystal structure: contains datablocks I, global. DOI: 10.1107/S1600536810036329/dn2600sup1.cif

Structure factors: contains datablocks I. DOI: 10.1107/S1600536810036329/dn2600Isup2.hkl

Additional supplementary materials:  crystallographic information; 3D view; checkCIF report

## Figures and Tables

**Table 1:** Hydrogen-bond geometry (Å, °)

*D*—H⋯*A*	*D*—H	H⋯*A*	*D*⋯*A*	*D*—H⋯*A*
C1—H1*A*⋯O41	0.970 (14)	2.480 (13)	3.1698 (16)	127.9 (10)
O2—H2⋯O41	0.828 (17)	1.913 (17)	2.7365 (13)	173.0 (16)
C15—H15⋯O462	0.940 (15)	2.408 (13)	3.0231 (17)	122.8 (11)
C1—H1*B*⋯O2^i^	0.938 (13)	2.549 (12)	3.2306 (16)	129.8 (10)
N14—H14⋯N34^ii^	0.893 (19)	1.851 (19)	2.7301 (16)	167.8 (17)
C26—H26⋯N12^iii^	0.938 (14)	2.456 (14)	3.3118 (18)	151.6 (11)
C43—H43⋯O442^iv^	0.972 (18)	2.472 (16)	3.280 (2)	140.4 (13)

Symmetry codes: (i) 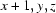; (ii) 
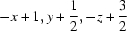
; (iii) 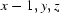; (iv) 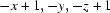.

**Fig. 1.**
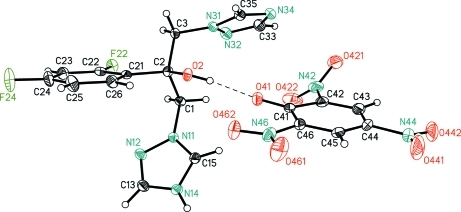
Anisotropic ellipsoid representation of the compound I together with atom labelling scheme. The ellipsoids are drawn at 50% probability level, hydrogen atoms are depicted as spheres with arbitrary radii and hydrogen bond between the ionic species is shown as dashed lines.

**Fig. 2.**
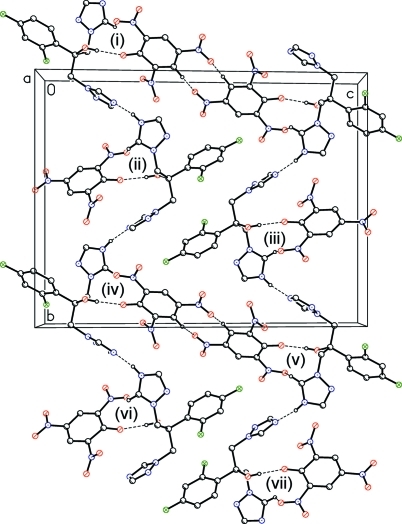
The crystal packing as seen approximately along [100] direction; hydrogen bonds are shown as dashed lines. Symmetry codes: (i) 1 - x,-y,1 - z; (ii) x,1/2 - y,-1/2 + z; (iii) 1 - x,1/2 + y,3/2 - z; (iv) 1 - x,1 - y,1 - z; (v) x,1 + y,z; (vi) x,3/2 - y,-1/2 + z; (vii) 1 - x,3/2+y,3/2 - z.

## supplementary crystallographic information

### Comment

Fluconazole is a synthetic antifungal agent that can be used for the treatment of a variety of *Candida albicans* and other fungal infections. It is currently the most widely used antifungal drug for maintenance therapy because it can be given orally, lacks major side effects, penetrates the central nervous system, and has broad efficiacy against most pathogenic yeasts, including *Cryptococcus neoformans* (Brammer *et al.*, 1990). The preparation and crystal characterization of one of the polymorphs, a monohydrate, and an ethyl acetate solvate of the fluconazole is reported (Caira *et al.*, 2003).

In the Cambridge Structural Database (Allen, 2002; version 5.31) there are 33 crystal structures of the metal complexes involving coordinated fluconazole molecule, three mentioned above structures of neutral fluconazole (including two solvates, a hydrate and an ethyl acetate), but the structure reported here is the first report on the protonated fluconazole, fluconazolium picrate (hereinafter referred to as 1, Scheme 1).

Figure 1 shows the perspective view of both charged components of 1. The fluconazolium cation is asymmetric (despite the possible symmetric conformation); both triazole rings are differently orientated with respect to the phenyl ring. The torsion angles C21—C2—N1—N11 and C21—C2—N3—N31 are -58.50 (13)° and 175.23 (10)°, respectively. The overall conformation of the cation might be described by mutual orientation of three planar fragments, a triazolinium ring (A), a 2,4-difluorophenyl ring (B) and the triazole ring (C). All these fragments are approximately planar, the largest deviations from the planarity are, maybe unexpectedly, obeserved within the phenyl ring (0.0120 (9) Å). The least squares planes make the dihedral angles of 52.51 (5)° (A/B), 24.53 (7)° (B/C) and 69.70 (6)° (A/C). As in the neutral fluconazole (Caira *et al.*, 2003) all these planes are approximately (within *ca* 25°) parallel to the central C—O bond. In the picrate anion, the six-membered ring is within 0.0082 (10)Å planar, and two of three nitro groups are almost coplanar with the ring plane (dihedral angles of 6.5 (2)° for the NO_2_ group *para* with respect to the C=O group, and 3.8 (2)° for one of the *ortho* groups), while the other *ortho*- group is significantly twisted, by an angle of 46.79 (13)°. The C—O bond of 1.2529 (15) Å, is longer than the typical C=O double bond, reflecting the anionic character of this, more appropriately described as C=O(-), bond.

The place of protonation is unequivocaly determined as N14. It is proved by (i) the location of the hydrogen atom in the difference Fourier map, and its subsequent succesful refinement, as well as by (ii) the geometrical characteristics of the nitrogen atoms.

The ionic components are joined by the O—H···O hydrogen bond and additionally by secondary - but relatively short *C*—*H*···*O*(nitro) contact (Figure 1). The geometrical details of hydrogen bonds are given in Table 1. In the crystal structure the cations are organized into the chains along the b-direction by means of N—H···N and C—H···O hydrogen bonds. The main structural motif is therefore a helix of cations with the anions attached subsequently to both sides of the helix (Fig. 2). It might be noted that the neighboring helical chains (which elements are related by 2_1_ screw along *y*) of different screwness, are only loosely connected, by C—H···O hydrogen bonds between the picrate anions(Fig. 3).

### Experimental

The title compound was synthesized by mixing equimolar amounts (1:1) of fluconazole (2 mmol) in 10 ml me thanol and picric acid (2 mmol) in 10 ml of methanol. The solution was stirred well and allowed to evaporate slowly at room temperature. Crystals suitable for single-crystal X-ray diffraction were grown from methanol solvent. The melting range was found to be 409 - 412 K.

### Refinement

Hydrogen atoms were located in the difference Fourier maps and isotropically refined.

### Crystal data

**Table:**

C_13_H_13_F_2_N_6_O^+^·C_6_H_2_N_3_O_7_^−^	*F*(000) = 1096
*M**_r_* = 535.40	*D*_x_ = 1.613 Mg m^−^^3^
Monoclinic, *P*2_1_/*c*	Mo *K*α radiation, λ = 0.71073 Å
Hall symbol: -P 2ybc	Cell parameters from 4361 reflections
*a* = 5.647 (1) Å	θ = 3–22°
*b* = 17.347 (2) Å	µ = 0.14 mm^−^^1^
*c* = 22.571 (2) Å	*T* = 100 K
β = 94.27 (1)°	Block, colourless
*V* = 2204.9 (5) Å^3^	0.2 × 0.2 × 0.15 mm
*Z* = 4	

### Data collection

**Table:**

Kuma KM-4-CCD four-circle diffractometer	3576 reflections with *I* > 2σ(*I*)
Radiation source: fine-focus sealed tube	*R*_int_ = 0.023
graphite	θ_max_ = 29.1°, θ_min_ = 3.0°
ω scan	*h* = −7→7
18260 measured reflections	*k* = −20→22
5165 independent reflections	*l* = −30→29

### Refinement

**Table:**

Refinement on *F*^2^	Primary atom site location: structure-invariant direct methods
Least-squares matrix: full	Secondary atom site location: difference Fourier map
*R*[*F*^2^ > 2σ(*F*^2^)] = 0.035	Hydrogen site location: inferred from neighbouring sites
*wR*(*F*^2^) = 0.082	All H-atom parameters refined
*S* = 0.99	*w* = 1/[σ^2^(*F*_o_^2^) + (0.045*P*)^2^] where *P* = (*F*_o_^2^ + 2*F*_c_^2^)/3
5165 reflections	(Δ/σ)_max_ = 0.004
403 parameters	Δρ_max_ = 0.25 e Å^−^^3^
0 restraints	Δρ_min_ = −0.24 e Å^−^^3^

### Special details

**Table:**

Geometry. All e.s.d.'s (except the e.s.d. in the dihedral angle between two l.s. planes) are estimated using the full covariance matrix. The cell e.s.d.'s are taken into account individually in the estimation of e.s.d.'s in distances, angles and torsion angles; correlations between e.s.d.'s in cell parameters are only used when they are defined by crystal symmetry. An approximate (isotropic) treatment of cell e.s.d.'s is used for estimating e.s.d.'s involving l.s. planes.
Refinement. Refinement of *F*^2^ against ALL reflections. The weighted *R*-factor *wR* and goodness of fit *S* are based on *F*^2^, conventional *R*-factors *R* are based on *F*, with *F* set to zero for negative *F*^2^. The threshold expression of *F*^2^ > σ(*F*^2^) is used only for calculating *R*-factors(gt) *etc*. and is not relevant to the choice of reflections for refinement. *R*-factors based on *F*^2^ are statistically about twice as large as those based on *F*, and *R*- factors based on ALL data will be even larger.

### Fractional atomic coordinates and isotropic or equivalent isotropic displacement parameters (Å^2^)

**Table:**

	*x*	*y*	*z*	*U*_iso_*/*U*_eq_	
C1	0.8252 (2)	0.11241 (8)	0.86298 (6)	0.0140 (3)	
H1A	0.824 (2)	0.0864 (9)	0.8249 (6)	0.016 (3)*	
H1B	0.965 (2)	0.1014 (8)	0.8865 (6)	0.009 (3)*	
N11	0.81990 (17)	0.19482 (7)	0.85106 (4)	0.0137 (2)	
N12	0.91454 (19)	0.24730 (7)	0.89174 (5)	0.0196 (3)	
C13	0.8628 (2)	0.31378 (10)	0.86711 (6)	0.0202 (3)	
H13	0.905 (3)	0.3619 (11)	0.8832 (7)	0.030 (4)*	
N14	0.74046 (18)	0.30535 (7)	0.81341 (5)	0.0171 (3)	
H14	0.679 (3)	0.3395 (11)	0.7870 (8)	0.039 (5)*	
C15	0.7157 (2)	0.23020 (9)	0.80471 (6)	0.0161 (3)	
H15	0.632 (2)	0.2046 (9)	0.7730 (7)	0.022 (4)*	
C2	0.6062 (2)	0.08837 (8)	0.89546 (5)	0.0134 (3)	
H2	0.416 (3)	0.0969 (11)	0.8248 (8)	0.035 (5)*	
O2	0.39596 (14)	0.10601 (6)	0.86009 (4)	0.0158 (2)	
C21	0.5940 (2)	0.13196 (8)	0.95392 (5)	0.0132 (3)	
C22	0.7674 (2)	0.12590 (8)	1.00064 (5)	0.0145 (3)	
F22	0.95997 (12)	0.08001 (5)	0.99397 (3)	0.01995 (19)	
C23	0.7581 (2)	0.16294 (9)	1.05430 (6)	0.0192 (3)	
H23	0.875 (2)	0.1575 (9)	1.0836 (6)	0.014 (3)*	
C24	0.5627 (2)	0.20856 (9)	1.06083 (6)	0.0235 (3)	
F24	0.54335 (15)	0.24419 (6)	1.11362 (4)	0.0393 (3)	
C25	0.3861 (2)	0.21871 (10)	1.01644 (6)	0.0237 (3)	
H25	0.256 (3)	0.2508 (9)	1.0228 (6)	0.023 (4)*	
C26	0.4056 (2)	0.18052 (9)	0.96320 (6)	0.0183 (3)	
H26	0.288 (2)	0.1884 (9)	0.9323 (6)	0.017 (4)*	
C3	0.6093 (2)	0.00119 (8)	0.90877 (5)	0.0159 (3)	
H3B	0.749 (2)	−0.0154 (9)	0.9330 (6)	0.022 (4)*	
H3A	0.461 (2)	−0.0115 (9)	0.9300 (6)	0.026 (4)*	
N31	0.60104 (17)	−0.04584 (7)	0.85546 (4)	0.0152 (2)	
N32	0.80503 (19)	−0.07447 (7)	0.83446 (5)	0.0204 (3)	
C33	0.7233 (2)	−0.11090 (9)	0.78608 (6)	0.0209 (3)	
H33	0.822 (2)	−0.1390 (10)	0.7617 (6)	0.025 (4)*	
N34	0.48410 (19)	−0.10769 (7)	0.77458 (5)	0.0195 (3)	
C35	0.4149 (2)	−0.06607 (9)	0.81927 (5)	0.0174 (3)	
H35	0.266 (3)	−0.0524 (9)	0.8276 (6)	0.023 (4)*	
C41	0.4057 (2)	0.08645 (9)	0.69270 (5)	0.0187 (3)	
O41	0.49841 (15)	0.08294 (6)	0.74481 (4)	0.0215 (2)	
C42	0.4803 (3)	0.03606 (9)	0.64625 (6)	0.0244 (3)	
N42	0.6721 (2)	−0.01813 (9)	0.66116 (5)	0.0302 (3)	
O421	0.6440 (2)	−0.08584 (7)	0.64488 (5)	0.0382 (3)	
O422	0.85228 (19)	0.00575 (7)	0.68870 (5)	0.0404 (3)	
C43	0.3793 (3)	0.03392 (11)	0.58915 (7)	0.0343 (4)	
H43	0.440 (3)	−0.0046 (11)	0.5629 (7)	0.037 (5)*	
C44	0.1963 (3)	0.08515 (10)	0.57331 (6)	0.0337 (4)	
N44	0.0919 (3)	0.08703 (10)	0.51239 (6)	0.0498 (5)	
O441	−0.0825 (4)	0.12829 (10)	0.50103 (6)	0.0829 (6)	
O442	0.1825 (2)	0.04704 (9)	0.47547 (5)	0.0610 (5)	
C45	0.1132 (3)	0.13529 (10)	0.61371 (6)	0.0278 (4)	
H45	−0.013 (3)	0.1690 (11)	0.6027 (7)	0.031 (4)*	
C46	0.2145 (2)	0.13681 (9)	0.67108 (6)	0.0204 (3)	
N46	0.11696 (19)	0.19341 (8)	0.70967 (5)	0.0229 (3)	
O461	−0.05047 (19)	0.23362 (8)	0.68997 (5)	0.0437 (3)	
O462	0.20457 (16)	0.20171 (7)	0.76060 (4)	0.0285 (3)	

### Atomic displacement parameters (Å^2^)

**Table:**

	*U*^11^	*U*^22^	*U*^33^	*U*^12^	*U*^13^	*U*^23^
C1	0.0138 (6)	0.0110 (7)	0.0171 (6)	0.0019 (5)	−0.0003 (5)	0.0008 (6)
N11	0.0122 (5)	0.0143 (6)	0.0146 (5)	−0.0004 (5)	0.0004 (4)	0.0002 (5)
N12	0.0211 (6)	0.0169 (7)	0.0198 (6)	−0.0036 (5)	−0.0053 (4)	−0.0005 (5)
C13	0.0238 (7)	0.0162 (8)	0.0199 (7)	−0.0017 (6)	−0.0037 (5)	−0.0002 (6)
N14	0.0185 (6)	0.0153 (7)	0.0170 (5)	0.0017 (5)	−0.0015 (4)	0.0033 (5)
C15	0.0162 (6)	0.0168 (8)	0.0151 (6)	−0.0008 (6)	−0.0006 (5)	0.0010 (6)
C2	0.0119 (6)	0.0147 (8)	0.0133 (6)	0.0009 (5)	−0.0020 (4)	0.0013 (5)
O2	0.0129 (4)	0.0220 (6)	0.0121 (4)	0.0011 (4)	−0.0023 (3)	−0.0008 (4)
C21	0.0130 (6)	0.0121 (7)	0.0147 (6)	−0.0036 (5)	0.0014 (4)	0.0008 (5)
C22	0.0134 (6)	0.0112 (8)	0.0191 (6)	−0.0003 (5)	0.0014 (5)	0.0025 (5)
F22	0.0174 (4)	0.0204 (5)	0.0211 (4)	0.0064 (3)	−0.0048 (3)	−0.0015 (3)
C23	0.0172 (7)	0.0225 (9)	0.0170 (6)	−0.0023 (6)	−0.0038 (5)	0.0008 (6)
C24	0.0258 (7)	0.0251 (10)	0.0197 (7)	−0.0018 (6)	0.0022 (5)	−0.0099 (6)
F24	0.0361 (5)	0.0550 (7)	0.0259 (4)	0.0109 (5)	−0.0038 (4)	−0.0226 (5)
C25	0.0177 (7)	0.0251 (9)	0.0283 (7)	0.0050 (6)	0.0007 (5)	−0.0084 (7)
C26	0.0149 (7)	0.0202 (9)	0.0194 (6)	−0.0011 (6)	−0.0027 (5)	−0.0016 (6)
C3	0.0198 (7)	0.0151 (8)	0.0123 (6)	−0.0018 (6)	−0.0016 (5)	−0.0007 (6)
N31	0.0161 (5)	0.0136 (7)	0.0156 (5)	−0.0010 (5)	0.0000 (4)	−0.0002 (5)
N32	0.0186 (6)	0.0182 (7)	0.0245 (6)	0.0024 (5)	0.0017 (4)	−0.0030 (5)
C33	0.0240 (7)	0.0172 (8)	0.0217 (7)	0.0015 (6)	0.0027 (5)	−0.0026 (6)
N34	0.0233 (6)	0.0161 (7)	0.0189 (6)	−0.0032 (5)	0.0000 (4)	−0.0015 (5)
C35	0.0176 (7)	0.0167 (8)	0.0177 (6)	−0.0020 (6)	0.0005 (5)	0.0001 (6)
C41	0.0228 (7)	0.0180 (8)	0.0157 (6)	−0.0094 (6)	0.0036 (5)	0.0012 (6)
O41	0.0247 (5)	0.0227 (6)	0.0165 (5)	−0.0004 (4)	−0.0019 (4)	−0.0013 (4)
C42	0.0338 (8)	0.0197 (9)	0.0209 (7)	−0.0073 (7)	0.0102 (6)	0.0005 (6)
N42	0.0391 (8)	0.0252 (9)	0.0291 (7)	−0.0068 (7)	0.0202 (6)	0.0009 (6)
O421	0.0653 (8)	0.0211 (7)	0.0307 (6)	−0.0015 (6)	0.0194 (5)	−0.0041 (5)
O422	0.0281 (6)	0.0334 (8)	0.0617 (8)	−0.0049 (5)	0.0165 (5)	0.0012 (6)
C43	0.0612 (11)	0.0253 (10)	0.0181 (7)	−0.0176 (9)	0.0147 (7)	−0.0043 (7)
C44	0.0629 (11)	0.0245 (10)	0.0130 (7)	−0.0170 (8)	−0.0031 (6)	0.0042 (7)
N44	0.0945 (13)	0.0355 (11)	0.0178 (7)	−0.0309 (10)	−0.0070 (7)	0.0025 (7)
O441	0.1649 (16)	0.0369 (10)	0.0378 (8)	0.0117 (11)	−0.0534 (9)	0.0016 (7)
O442	0.0873 (10)	0.0801 (12)	0.0165 (6)	−0.0488 (9)	0.0104 (6)	−0.0097 (7)
C45	0.0388 (9)	0.0231 (10)	0.0204 (7)	−0.0132 (7)	−0.0053 (6)	0.0088 (7)
C46	0.0247 (7)	0.0213 (9)	0.0151 (6)	−0.0082 (6)	0.0005 (5)	0.0010 (6)
N46	0.0196 (6)	0.0293 (8)	0.0193 (6)	−0.0016 (5)	−0.0012 (4)	0.0051 (6)
O461	0.0400 (6)	0.0577 (10)	0.0319 (6)	0.0229 (6)	−0.0079 (5)	0.0049 (6)
O462	0.0275 (5)	0.0390 (8)	0.0183 (5)	0.0075 (5)	−0.0022 (4)	−0.0036 (5)

### Geometric parameters (Å, °)

**Table:**

C1—N11	1.4546 (18)	C3—H3B	0.971 (14)
C1—C2	1.5414 (17)	C3—H3A	1.018 (14)
C1—H1A	0.970 (14)	N31—C35	1.3297 (16)
C1—H1B	0.938 (13)	N31—N32	1.3710 (15)
N11—C15	1.3136 (16)	N32—C33	1.3152 (18)
N11—N12	1.3728 (16)	C33—N34	1.3577 (18)
N12—C13	1.304 (2)	C33—H33	0.945 (15)
C13—N14	1.3577 (17)	N34—C35	1.3230 (18)
C13—H13	0.934 (18)	C35—H35	0.909 (14)
N14—C15	1.3240 (19)	C41—O41	1.2529 (15)
N14—H14	0.893 (19)	C41—C46	1.445 (2)
C15—H15	0.940 (15)	C41—C42	1.451 (2)
C2—O2	1.4140 (14)	C42—C43	1.370 (2)
C2—C21	1.5267 (17)	C42—N42	1.454 (2)
C2—C3	1.542 (2)	N42—O422	1.2245 (17)
O2—H2	0.828 (17)	N42—O421	1.2373 (18)
C21—C26	1.3850 (19)	C43—C44	1.390 (3)
C21—C22	1.3886 (17)	C43—H43	0.972 (18)
C22—F22	1.3652 (15)	C44—C45	1.368 (2)
C22—C23	1.3753 (19)	C44—N44	1.456 (2)
C23—C24	1.375 (2)	N44—O442	1.225 (2)
C23—H23	0.904 (13)	N44—O441	1.229 (2)
C24—F24	1.3539 (16)	C45—C46	1.3766 (19)
C24—C25	1.371 (2)	C45—H45	0.942 (17)
C25—C26	1.384 (2)	C46—N46	1.4483 (19)
C25—H25	0.940 (15)	N46—O462	1.2257 (14)
C26—H26	0.938 (14)	N46—O461	1.2310 (16)
C3—N31	1.4515 (16)		
			
N11—C1—C2	110.35 (10)	N31—C3—C2	113.00 (10)
N11—C1—H1A	107.1 (9)	N31—C3—H3B	105.9 (9)
C2—C1—H1A	110.0 (8)	C2—C3—H3B	113.4 (10)
N11—C1—H1B	108.0 (9)	N31—C3—H3A	107.0 (9)
C2—C1—H1B	110.4 (8)	C2—C3—H3A	107.8 (9)
H1A—C1—H1B	111.0 (11)	H3B—C3—H3A	109.5 (12)
C15—N11—N12	110.59 (12)	C35—N31—N32	109.77 (11)
C15—N11—C1	127.48 (11)	C35—N31—C3	129.25 (11)
N12—N11—C1	121.76 (10)	N32—N31—C3	120.95 (10)
C13—N12—N11	103.75 (11)	C33—N32—N31	102.08 (10)
N12—C13—N14	111.61 (14)	N32—C33—N34	114.93 (12)
N12—C13—H13	125.6 (10)	N32—C33—H33	123.1 (9)
N14—C13—H13	122.7 (10)	N34—C33—H33	121.9 (9)
C15—N14—C13	106.23 (12)	C35—N34—C33	102.89 (11)
C15—N14—H14	121.5 (12)	N34—C35—N31	110.32 (12)
C13—N14—H14	132.2 (12)	N34—C35—H35	129.1 (9)
N11—C15—N14	107.81 (12)	N31—C35—H35	120.6 (9)
N11—C15—H15	123.8 (10)	O41—C41—C46	126.56 (13)
N14—C15—H15	128.3 (10)	O41—C41—C42	121.71 (14)
O2—C2—C21	106.91 (10)	C46—C41—C42	111.71 (12)
O2—C2—C1	110.03 (10)	C43—C42—C41	124.96 (15)
C21—C2—C1	111.50 (11)	C43—C42—N42	116.77 (14)
O2—C2—C3	108.61 (10)	C41—C42—N42	118.25 (12)
C21—C2—C3	108.55 (10)	O422—N42—O421	123.71 (15)
C1—C2—C3	111.11 (11)	O422—N42—C42	118.37 (14)
C2—O2—H2	109.4 (11)	O421—N42—C42	117.92 (13)
C26—C21—C22	115.93 (12)	C42—C43—C44	118.30 (16)
C26—C21—C2	121.10 (11)	C42—C43—H43	116.8 (9)
C22—C21—C2	122.97 (11)	C44—C43—H43	124.9 (9)
F22—C22—C23	116.91 (11)	C45—C44—C43	121.37 (14)
F22—C22—C21	118.86 (11)	C45—C44—N44	118.70 (17)
C23—C22—C21	124.22 (12)	C43—C44—N44	119.92 (16)
C24—C23—C22	116.55 (12)	O442—N44—O441	123.84 (15)
C24—C23—H23	122.1 (9)	O442—N44—C44	117.99 (19)
C22—C23—H23	121.4 (9)	O441—N44—C44	118.18 (17)
F24—C24—C25	118.83 (13)	C44—C45—C46	119.99 (17)
F24—C24—C23	118.39 (12)	C44—C45—H45	120.7 (10)
C25—C24—C23	122.78 (13)	C46—C45—H45	119.3 (10)
C24—C25—C26	118.19 (13)	C45—C46—C41	123.64 (14)
C24—C25—H25	119.9 (9)	C45—C46—N46	115.37 (14)
C26—C25—H25	121.9 (9)	C41—C46—N46	121.00 (11)
C25—C26—C21	122.29 (12)	O462—N46—O461	121.17 (13)
C25—C26—H26	118.5 (9)	O462—N46—C46	119.90 (12)
C21—C26—H26	119.2 (9)	O461—N46—C46	118.91 (11)
			
C2—C1—N11—C15	−87.38 (14)	C35—N31—N32—C33	−0.22 (15)
C2—C1—N11—N12	87.44 (13)	C3—N31—N32—C33	−178.32 (12)
C15—N11—N12—C13	−0.40 (13)	N31—N32—C33—N34	0.08 (16)
C1—N11—N12—C13	−176.01 (11)	N32—C33—N34—C35	0.09 (17)
N11—N12—C13—N14	0.19 (14)	C33—N34—C35—N31	−0.23 (16)
N12—C13—N14—C15	0.09 (15)	N32—N31—C35—N34	0.30 (16)
N12—N11—C15—N14	0.47 (13)	C3—N31—C35—N34	178.20 (13)
C1—N11—C15—N14	175.77 (11)	O41—C41—C42—C43	176.56 (14)
C13—N14—C15—N11	−0.34 (13)	C46—C41—C42—C43	−1.9 (2)
N11—C1—C2—O2	59.95 (14)	O41—C41—C42—N42	−1.6 (2)
N11—C1—C2—C21	−58.50 (13)	C46—C41—C42—N42	179.93 (12)
N11—C1—C2—C3	−179.73 (10)	C43—C42—N42—O422	133.86 (14)
O2—C2—C21—C26	−2.59 (17)	C41—C42—N42—O422	−47.81 (17)
C1—C2—C21—C26	117.71 (13)	C43—C42—N42—O421	−45.70 (18)
C3—C2—C21—C26	−119.58 (13)	C41—C42—N42—O421	132.63 (13)
O2—C2—C21—C22	177.43 (12)	C41—C42—C43—C44	2.1 (2)
C1—C2—C21—C22	−62.27 (17)	N42—C42—C43—C44	−179.72 (14)
C3—C2—C21—C22	60.44 (16)	C42—C43—C44—C45	−1.7 (2)
C26—C21—C22—F22	−178.86 (11)	C42—C43—C44—N44	177.23 (14)
C2—C21—C22—F22	1.12 (19)	C45—C44—N44—O442	173.29 (15)
C26—C21—C22—C23	1.7 (2)	C43—C44—N44—O442	−5.7 (2)
C2—C21—C22—C23	−178.30 (13)	C45—C44—N44—O441	−7.0 (2)
F22—C22—C23—C24	−179.51 (12)	C43—C44—N44—O441	174.00 (17)
C21—C22—C23—C24	−0.1 (2)	C43—C44—C45—C46	1.4 (2)
C22—C23—C24—F24	178.05 (13)	N44—C44—C45—C46	−177.58 (14)
C22—C23—C24—C25	−1.2 (2)	C44—C45—C46—C41	−1.3 (2)
F24—C24—C25—C26	−178.53 (14)	C44—C45—C46—N46	178.46 (13)
C23—C24—C25—C26	0.7 (2)	O41—C41—C46—C45	−176.88 (14)
C24—C25—C26—C21	1.1 (2)	C42—C41—C46—C45	1.5 (2)
C22—C21—C26—C25	−2.2 (2)	O41—C41—C46—N46	3.3 (2)
C2—C21—C26—C25	177.80 (14)	C42—C41—C46—N46	−178.31 (12)
O2—C2—C3—N31	59.34 (13)	C45—C46—N46—O462	−176.14 (13)
C21—C2—C3—N31	175.23 (10)	C41—C46—N46—O462	3.67 (19)
C1—C2—C3—N31	−61.82 (13)	C45—C46—N46—O461	2.31 (19)
C2—C3—N31—C35	−81.53 (16)	C41—C46—N46—O461	−177.88 (13)
C2—C3—N31—N32	96.16 (14)		

### Hydrogen-bond geometry (Å, °)

**Table:**

*D*—H···*A*	*D*—H	H···*A*	*D*···*A*	*D*—H···*A*
C1—H1A···O41	0.970 (14)	2.480 (13)	3.1698 (16)	127.9 (10)
O2—H2···O41	0.828 (17)	1.913 (17)	2.7365 (13)	173.0 (16)
C15—H15···O462	0.940 (15)	2.408 (13)	3.0231 (17)	122.8 (11)
C1—H1B···O2^i^	0.938 (13)	2.549 (12)	3.2306 (16)	129.8 (10)
N14—H14···N34^ii^	0.893 (19)	1.851 (19)	2.7301 (16)	167.8 (17)
C26—H26···N12^iii^	0.938 (14)	2.456 (14)	3.3118 (18)	151.6 (11)
C43—H43···O442^iv^	0.972 (18)	2.472 (16)	3.280 (2)	140.4 (13)

Symmetry codes: (i) *x*+1, *y*, *z*; (ii) −*x*+1, *y*+1/2, −*z*+3/2; (iii) *x*−1, *y*, *z*; (iv) −*x*+1, −*y*, −*z*+1.

## References

[bb1] Allen, F. H. (2002). *Acta Cryst.* B**58**, 380–388. 10.1107/s0108768102003890 12037359

[bb2] Altomare, A., Cascarano, G., Giacovazzo, C. & Guagliardi, A. (1993). *J. Appl. Cryst.* **26**, 343–350.

[bb3] Brammer, K. W., Farrow, P. R. & Faulkner, J. K. (1990). *Rev* *Infect* *Dis* **12**, S318–S326. 10.1093/clinids/12.supplement_3.s318 2184510

[bb4] Caira, M. R., Alkhamis, A. & Obaidat, R. M. (2003). *J. Pharm. Sci.* **93**, 601–611. 10.1002/jps.10541 14762899

[bb5] Oxford Diffraction (2009). *CrysAlis PRO* Oxford Diffraction Ltd, Yarnton, England.

[bb6] Sheldrick, G. M. (2008). *Acta Cryst.* A**64**, 112–122. 10.1107/S0108767307043930 18156677

[bb7] Siemens (1989). *Stereochemical Workstation Operation Manual.* Siemens Analytical X-ray Instruments Inc., Madison, Wisconsin, USA.

